# Silence of cancer susceptibility candidate 9 inhibits gastric cancer and reverses chemoresistance

**DOI:** 10.18632/oncotarget.14871

**Published:** 2017-01-27

**Authors:** Chao Shang, Lin Sun, Jiale Zhang, Bochao Zhao, Xiuxiu Chen, Huimian Xu, Baojun Huang

**Affiliations:** ^1^ Department of Neurobiology, College of Basic Medicine, China Medical University, Shenyang, 110001, China; ^2^ Department of Surgical Oncology, First Affiliated Hospital, China Medical University, Shenyang, 110004, China; ^3^ Department of Gastrointestinal Surgery, Dalian Municipal Central Hospital, Dalian, 116033, China

**Keywords:** long noncoding RNA, CASC9, gastric cancer, malignancy, chemotherapy resistance

## Abstract

Cancer Susceptibility Candidate 9 (CASC9) is a novel gene generating long non-coding RNA (lncRNA) with oncogenic potential that was first identified in esophageal cancer. In this study, we have found that CASC9 was overexpressed in gastric cancer (GC) compared to normal gastric tissue. A higher expression level was associated with aggressive pathological characteristics, including deep invasion, poor differentiation and lymph node metastases. In two gastric cancer cell lines, BGC823 and SGC7901, CASC9 were both overexpressed compared to that of normal gastric epithelial cell (GES-1). Moreover, the expression of CASC9 was even higher in BGC823/DR and SGC7901/DR cells that are resistant to paclitaxel or adriamycin. CASC9 knockdown inhibited proliferation and promoted cell apoptosis In BGC823/DR and SGC7901/DR cells. The invasion potential was also significantly inhibited measured by Transwell assay. In addition, CASC9 knockdown in BGC823/DR and SGC7901/DR cells restored chemosensitivity to paclitaxel and adriamycin. This was associated with decreased expression of multidrug resistance 1 (MDR1) protein. Taken together, our data suggest that expression of lncRNA CASC9 correlated with aggressive pathological characteristics of GC, it may serve as a potential oncogene to regulate proliferation, invasion, and chemoresistance of GC cells.

## INTRODUCTION

Gastric cancer (GC) is one of the most common solid malignant tumors [1]. Because locally advanced GC has high a frequency of micrometastasis and relapse, its prognosis is poor. The 5 years survival after radical surgery was 30%~50%. Perioperative and adjuvant chemotherapy have been shown to be able to decrease distant metastasis and improve survival by 10–15% [2–4]. Chemoresistance likely has contributed to the relapse after chemotherapy. It is crucial to understand the underlying mechanism of chemoresistance and find novel therapeutic targets to further improve the outcome of GC.

Long noncoding RNA (lncRNA) is a type of endogenous RNA that is not translated to protein. Over the past ten years, the research in lncRNA has progressed rapidly. LncRNAs could act as transcriptional regulators for many genes, and participate in key steps of cancer proliferation, invasion and chemoresistance [5–7]. A few lncRNAs have been proposed to act as oncogenes or tumor suppressor genes in malignant tumors [8, 9].

Cancer Susceptibility Candidate 9 (CASC9) gene is located at 8q21.11, it encodes a lncRNA. The gene was first reported in esophageal squamous cell carcinoma (ESCC), where it regulates migration and invasion of the cancer cells [10]. By using microarray assays, we have found that CASC9 was expressed nearly eightfold higher in GC tissue than that of control normal gastric tissues. The observation has prompted us to further study its role in cell proliferation, invasion, and chemoresistance. Our findings suggest that CASC9 plays a significant role in these steps. CASC9 may serve as a therapeutic target of GC.

## RESULTS

### CASC9 was up-regulated in GC tissues and cell lines

**Figure 1:**
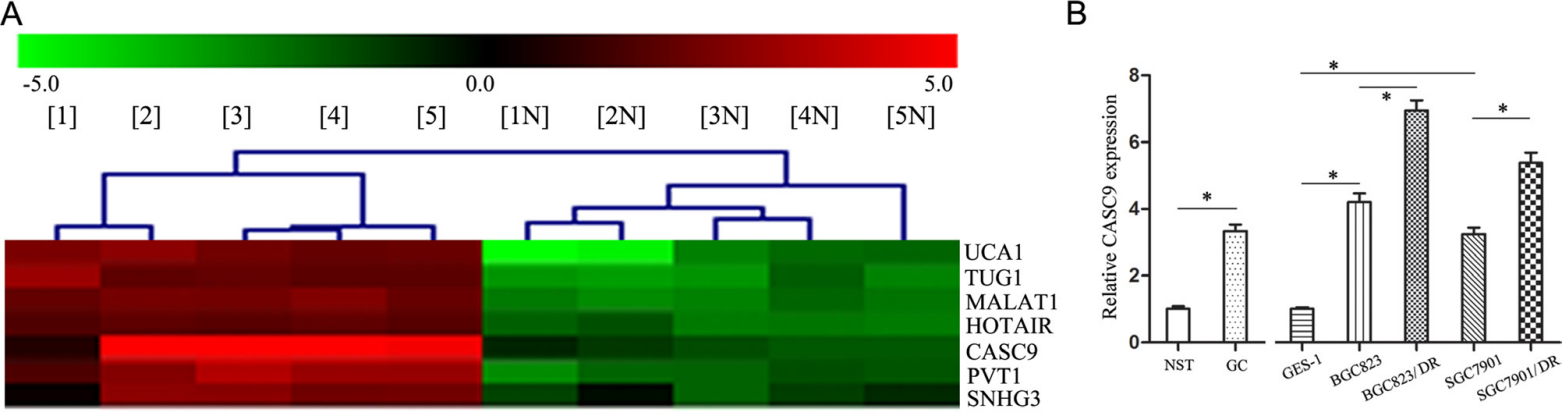
CASC9 is overexpressed in gastric cancer (**A**) Representative microarray analysis of CASC9 in five gastric cancer (GC) specimens and paired control normal stomach tissues (NST). 1–5: GC tissue, 1N-5N: NST. (**B**) The CASC9 expression levels examined by qRT-PCR in GC samples and NST samples (*n* = 89). And the CASC9 expression examined by qRT-PCR in BGC823, SGC7901, GES1 cells, as well as in BGC823/DR and SGC7901/DR cells.

By using microarray assays, we found that the expression of CASC9 was nearly eightfold higher in 5 GC specimens examined than that in paired control peritumoral specimens from the same patients (Figure 1A). This observation was further confirmed by qRT-PCR in 89 GC tissues, compared with control NST, the expression of CASC9 in 89 GC tissues was significantly higher (3.34 ± 0.24, *P <* 0.05, Figure 1B). The expression of CASC9 in gastric cancer cell lines, BGC823 and SGC7901 cells (4.21 ± 0.25, 3.24 ± 0.20), were higher than that in control normal gastric epithelial cells (GES-1) (*P <* 0.05, Figure 1B).

We have further analyzed the correlations between CASC9 expression and clinical pathological characteristics of GC. The expression levels of CASC9 were associated with increased tumor size, depth of invasion, and number of lymph node metastasis (*P <* 0.05, Table 1). The expression levels of CASC9 were much higher in tumors of Borrmann 3 and 4 types, or tumors with poor differentiation, INFγ-infiltrating pattern than in tumors of Borrman 1 and 2 type, or tumors with well or moderate differentiation, and INFα/β-infiltrating pattern (*P <* 0.05). These results suggest that CASC9 may play a role in tumor progression and metastasis of GC.

**Table 1:** The correlation between expression of CASC9 mRNA and clinical characteristics in 89 gastric cancer specimens

Factors		*n*	x ± s	F	*P*
size (cm)	< 6.0	41	2.880 ± 1.643		
	≥ 6.0	48	3.762 ± 1.966	4.916	0.029*
site	lower	48	3.656 ± 2.042		
	middle	19	3.286 ± 1.824		
	upper	16	2.129 ± 0.956	3.101	0.031*
	total	6	4.260 ± 0.784		
Borrmann type	1/2	15	2.063 ± 0.789	7.320	0.008*
	3/4	74	3.584 ± 1.914		
Depth of invassion	T2/3	25	2.612 ± 1.270	6.154	0.015*
	T4	64	3.684 ± 1.995		
infiltrating pattern	INFα/β	34	2.306 ± 1.093	21.579	< 0.001*
	INFγ	55	4.036 ± 1.956		
differentiation	Well/moderate	31	2.114 ± 1.147	23.782	< 0.001*
	poor	58	3.979 ± 1.858		
Lymphatic/venous invasion	–	54	3.362 ± 2.208	0.004	0.947
	+	35	3.389 ± 1.179		
TNM Node classification (7th)	N0 (0)	27	1.916 ± 1.066	21.219	< 0.001*
	N1(1–2)	23	3.258 ± 1.324		
	N2 (3–6)	21	3.670 ± 1.524		
	N3 (≥ 7)	18	5.359 ± 1.864		

Note: *indicates the difference is significant.

### CASC9 was up-regulated in GC drug-resistant cell lines

The IC50 of paclitaxel in BGC823/DR and SGC7901/DR cells were 183.33 ± 0.41 μg/L and 146.54 ± 0.57 μg/L, respectively, significantly higher than that in BGC823 and SGC7901 cells , 41.52 ± 0.27 μg/L and 33.28 ± 0.24 μg/L, respectively (*P <* 0.05). The calculated resistance indexs (RI) were 4.42 and 4.40.

The IC50 of adriamycin BGC823/DR and SGC7901/DR cells were 7.26 ± 0.21 μg/mL and 5.41 ± 0.14 μg/mL, respectively, significantly higher than that in BGC823 and SGC7901 cells 3.02 ± 0.18 μg/mL and 1.52 ± 0.13 μg/mL, respectively(*P <* 0.05). The IC50 in drug-resistant cells was much higher, their resistance index (RI) were 2.41 and 3.55.

The BGC823/DR and SGC7901/DR cells are resistant to paclitaxel and Adriamycin at tested levels. The expression of CASC9 expression were significantly higher in drug-resistant BGC823/DR and SGC7901/DR cells (6.95 ± 0.31, 5.38 ± 0.34, *P <* 0.05, Figure 1B). These results suggest that CASC9 overexpresion is associated chemoresistance of GC.

### CASC9 knockdown inhibited the growth and invasion of drug-resistant GC cells

**Figure 2:**
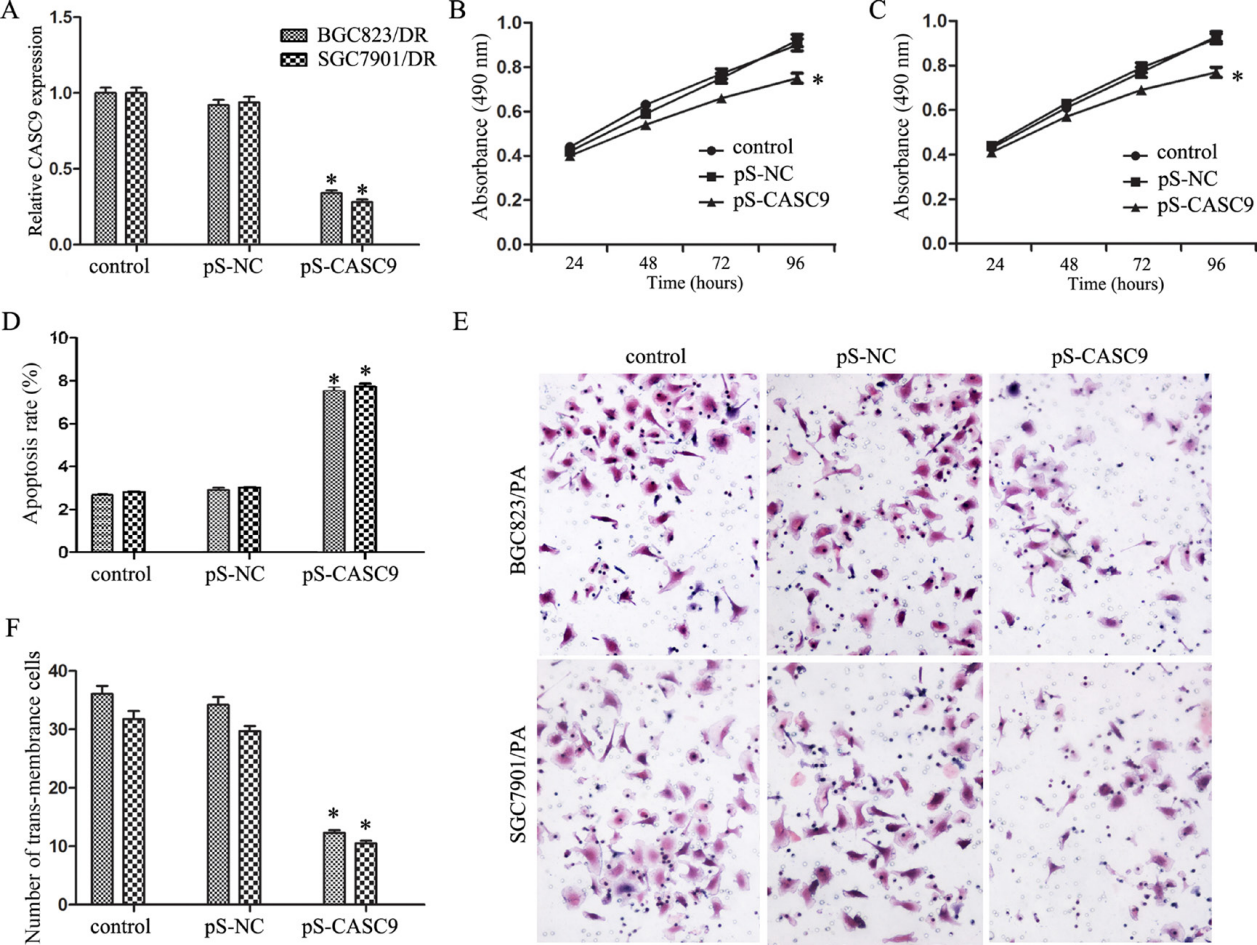
Knockdown of CASC9 inhibited proliferation and invasion (**A**) Expression level of CASC9 in BGC823/DR and SGC7901/DR cells after transfection of pS-CASC9. (**B**) Growth curve of BGC823/DR cells after CASC9 knockdown. (**C**) Growth curve of SGC7901/DR cells after CASC9 knockdown. (**D**) Apoptosis of BGC823/DR and SGC7901/DR cells after CASC9 knockdown. (**E**) Invasion assay by transwell analysis of BGC823/DR and SGC7901/DR cells after CASC9 knockdown. (**F**) CASC9 knockdown decreased the numbers of trans-membrance BGC823/DR and SGC7901/DR cells.

To evaluate whether manipulation of CASC9 will affect the growth and invasion of drug-resistant GC cells, cells stably expressing CASC9 silencing vector were established, and the knockdown was confirmed by qRT-PCR (*P <* 0.05, Figure 2A).

As shown in Figure 2B and 2C, CASC9 knockdown inhibited cell growth of both BGC823/DR and SGC7901/DR cells (*P <* 0.05). Flow cytometry revealed that CASC9 knockdown is associated with significantly increased apoptosis (Figure 2D, *P <* 0.05). As shown in Figure 2E and 2F, CASC9 knockdown in BGC823/DR and SGC7901/DR cells also impeded cell invasion (*P <* 0.05).

### CASC9 knockdown inhibited chemoresistance in drug-resistant GC cells

**Figure 3:**
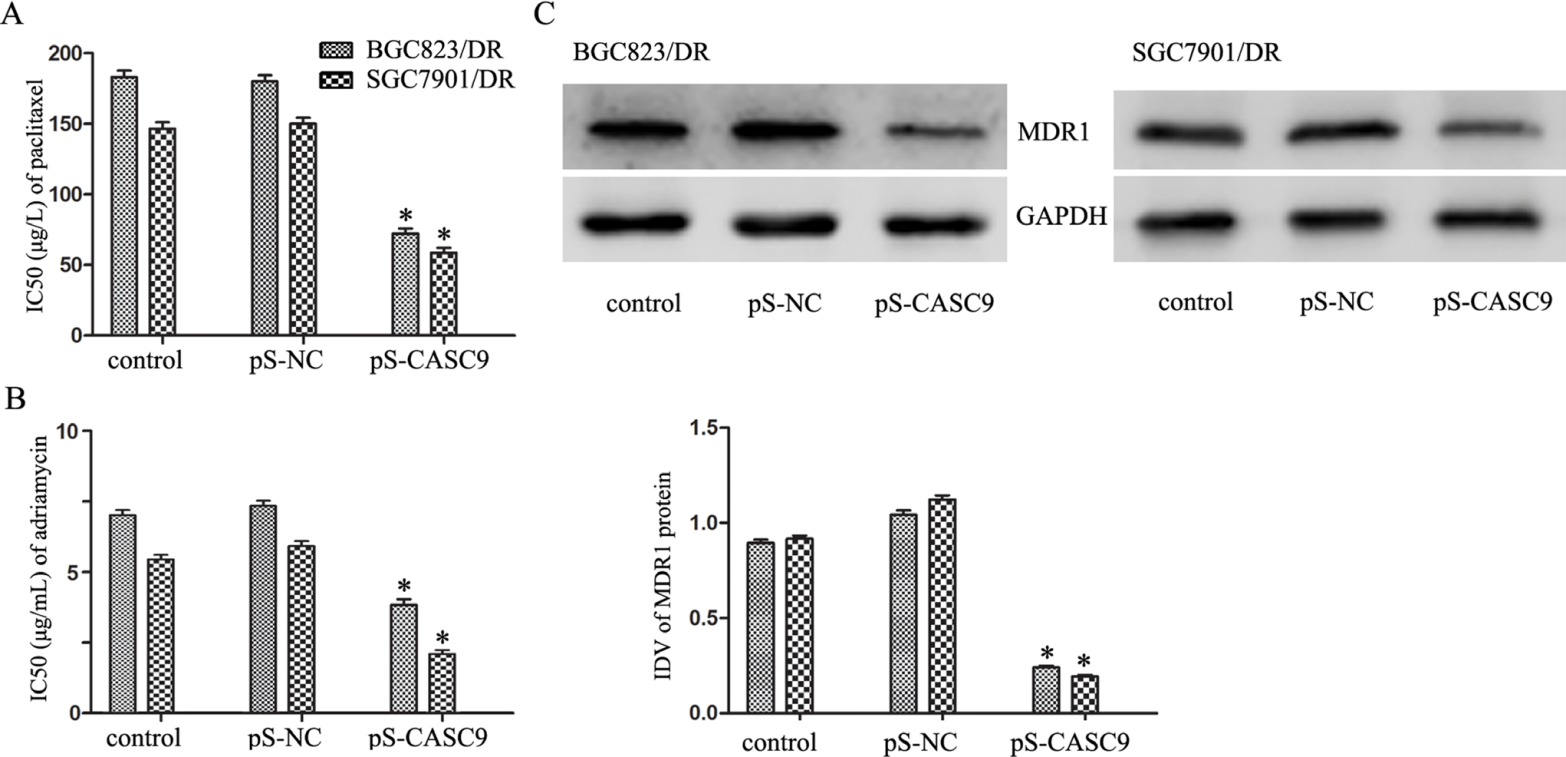
Knockdown of CASC9 reverses chemoresistance in BGC823/DR and SGC7901/DR cells (**A**) IC50 of BGC823/DR and SGC7901/DR cells to paclitaxel after CASC9 knockdown. (**B**) IC50 of BGC823/DR and SGC7901/DR cells to adriamycin after CASC9 knockdown. (**C**) The expression of MDR1 protein in BGC823/DR and SGC7901/DR cells after CASC9 knockdown.

CASC9 knockdown in BGC823/DR and SGC7901/DR cells resensitized them to paclitaxel: IC50 decreased from 183.33 ± 0.41 μg/L and 146.54 ± 0.57 μg/L to 72.31 ± 0.28 μg/L and 58.62 ± 0.32 μg/L, respectively (*P <* 0.05 Figure 3A). Similarly, CASC9 knockdown restored their sensitivities to Adriamycin, IC50 decreased from 7.26 ± 0.21 μg/mL and 5.41 ± 0.14 μg/mL to 4.17 ± 0.19 μg/mL and 2.28 ± 0.16 μg/mL, respectively (*P <* 0.05, Figure 3B).

### CASC9 knockdown is associated with downregulation of multidrug resistance 1 (MDR1) in drug-resistant GC cells

Based on the preliminary results of microarray assays (data not shown), a positive correlation between CASC9 and MDR1 was suggested. We tested the expression level of MDR1 in BGC823/DR and SGC7901/DR cells before and after CASC9 knockdown by Western blots. As a matter of fact, knockdown of CASC9 was associated decreased expression of MDR1 (Figure 3C, *P <* 0.05.

## DISCUSSION

CASC9 was originally found in ESCC by next-generation sequencing and bioinformatics analyses [11], higher expression level of CASC9 was found to be correlated with poor differentiation of ESCC [10]. Interestingly, high expression level of CASC9 was also found in GC specimen by microarray analysis. The overexpression remains to be true in the two well established gastric cell lines, BGC823 and SGC7901, but not in the normal gastric epithelial cells, suggesting that it may potentially promote cell growth. In fact, in GC tumor specimens, higher expression level of CASC9 correlated well with increased tumor size, depth of tumor invasion, and number of lymph node metastasis, Borrmann 3 or 4 subtypes, poor differentiation, and INFγ infiltrating pattern. Silencing the expression of CASC9 by pS-CASC9 transfection not only inhibited the proliferation, promoted apoptosis in BGC823/DR and SGC7901/DR cells, but also inhibited their invasion potential by Transwell assay. The underlying mechanism as to how CASC9 promote proliferation remains to be elucidated. LncRNA regulates the expression and function of downstream genes through a variety of ways. Li *P* et al. reported that lncRNA CASC2 suppresses the proliferation of gastric cancer cells by regulating the MAPK signaling pathway [12]. Our previous study showed that another lncRNA, TUSC7 (tumor suppressor candidate 7), was targeted and inhibited reciprocally by miR-23b, and acted as a tumor-suppressor gene in glioma cells [5]. We speculate that CASC9 might play its role in GC through regulating the expression of its target genes, our ongoing studies will shed light in the future on the underlying mechanisms.

The expression of CASC9 was further upregulated in drug-resistant BGC823/DR and SGC7901/DR cell lines, suggesting that CASC9 overexpression might be involved in chemoresistance of GC cells. As a matter of fact, knockdown of CASC9 significantly reduced chemoresistance to paclitaxel and Adriamycin in BGC823/DR and SGC7901/DR cell lines. The findings of a positive correlation between the level of CASC9 and MDR1 is interesting, it is not certain if CASC9 gene directly regulates MDR1 expression, further experiment will be needed to investigate the underlying mechanisms.

In conclusion, lncRNA CASC9 is frequently overexpressed in GC, it may promote cell growth and chemoresistance to paclitaxel and adriamycin in GC. Targeting CASC9 might provide therapeutic potential for GC.

## MATERIALS AND METHODS

### Clinical specimens

Total of 89 GC and paired control normal stomach tissues (NST) were obtained from the First Affiliated Hospital of China Medical University from Feb 2016 to Aug 2016. The tissue samples were stored in liquid nitrogen. The clinicopathological data was confirmed by pathologist. The Ethics Committees of China Medical University approved this study, and research consents were given by the patients before the study.

### Arraystar LncRNA array

Total RNA was extracted by the TRIzol^®^ Reagent from GC and NST tissues according to the operation manual. Then the total RNA was purified by the RNasey Mini Kit (Qiagen p/n 74104). Human 8 × 60K LncRNA expression array purchased from KangchengBio Corporation (Shanghai, China) was used for the determine the lncRNAs/mRNAs expression profiles. RNA was labeled with Quick Amp Labeling Kit One-Color (Agilent p/n 5190-0442), re-purified with RNeasy Mini Kit (Qiagen p/n 74104) and measured by NanoDrop ND-1000. Equal amount of RNA was then hybridized with Agilent Gene Expression Hybridization Kit (Agilent p/n 5188-5242). Following repeated washing, the arrays were scanned and the data were extracted by using Agilent Feature Extraction Software.

### Cell culture

Human gastric cancer BGC823 and SGC7901 cells and normal gastric epithelial cell GES-1 were purchased from China Academy of Chinese Medical Sciences. Drug-resistant BGC823/DR and SGC7901/DR were preserved in our laboratory. Those cells were cultured in RPMI-1640 medium containing 10% fetal bovine serum (Gibco, Carlsbad, CA, USA)at 37°C with 5% CO_2_ according to our previous study [13].

### Quantitative real time -PCR (qRT-PCR)

Total RNA was harvested with TRIzol reagent (Life Technologies Corporation, Carlsbad, CA, USA) and reverse transcribed to cDNA using Reverse-Transcription Kit (Applied Biosystems, USA). One Step SYBR RT-PCR Kit (TaKaRa, Japan) was used to detect the CASC9 expression in GC tissues and cell lines according to manufacturer's protocol. Relative CASC9 expression was quantified with the relative quantitative method relative to GAPDH level. The primers of CASC9 were 5′- AGATGAAGCCGGTACCTCAGAT -3′ (sense) and 5′- TCACTTTAAAGAGGGAGAGGAG -3′ (antisense).

### Vector construction and transfection

The silence vector pS-CASC9 and empty pSilencer (NC) were obtained from Fitgene Company (Guangzhou, China). pS-CASC9 and pS-NC were transfected with Lipofectamine 3000 Reagent (Invitrogen, Foster City, CA, USA) according to manufacturer's protocol after 24 h of culture. G418 (Invitrogen, Foster City, CA, USA) was used to select cell lines stably expressing the vectors, and qRT-PCR was used to confirm and measure the konckdown. Eight passages had been passed by the time of conducting the experiment.

### Proliferation and chemotherapy resistance assay

Cellular proliferation was measured by MTT assay according to our previous study [14]. Cells were seeded into 96-well plates with 3000 cells per well and treated with paclitaxel (10 μg/L, 25 μg/L, 50 μg/L, 100 μg/L and 200 μg/L) or adriamycin (0.005 μg/mL, 0.05 μg/mL, 0.5 μg/mL, 5 μg/mL, 50 μg/mL) 24 h later [13, 15]. After 48 h, the cellular viability was evaluated, and the dose-response curve was drawn to calculate the half maximal inhibitory concentration (IC50) using a Probit regression model.

### Cell invasion assay

Cell invasion assay was conducted by Transwell chamber (Costar, Corning, NY, USA) with polycarbonic membrane (6.5mmin diameter, 8 μm pore size) and Matrigel (BD, NJ, USA). The Transwell membrane was coated with 80 μL of Matrigel solution (500 ng/μL; BD, Franklin Lakes, NJ, USA) and incubated at 37°C for 4 h. The transfected cells were resuspended in 100 μL serum-free medium at a density of 5 × 10^5^ cells/mL and added in the upper chamber. 600 μL of DMEM/high-glucose or DMEM/F12 medium supplemented with 10% FBS was added to the lower chamber. After incubation for 48 h, cells above the membrane surface were mechanically removed. Cells that had invaded to the lower side of the membrane were fixed with methanol and stained with 20% Giemsa. Stained cells were counted under a microscope in five randomly chosen fields and the average number was calculated.

### Measurement of apoptosis

Cell apoptosis rate was quantified using Annexin V-FITC apoptosis detection kit (Biosci, Hangzhou, China) with flow cytometry according to manufacturer's protocol. Data was analyzed according to our previous report [5].

### Western blot analysis

Protein samples were prepared with SDS-PAGE gels electrophoresis and transferred to PVDF membranes. PVDF membranes were incubated with primary MDR1 antibody (ab129450, Abcam, USA), then incubated with horseradish peroxidase conjugated secondary antibody. Immunoblots were visualized by chemiluminescence detection kit (Gene, Hongkong, China). ImageJ software (BD, Franklin Lakes, NJ, USA) was used to quantify the level of protein expression by calculating integrated density value (IDV).

### Statistical analysis

All data were showed as means ± SEM of five independent experiments and analyzed with GraphPad Prism 5.0 (Graphpad Software, La Jolla, CA). Student's *t*-test was used to analyze the difference between experiments. The association between CASC9 expression and pathological characteristics were analyzed by one-way ANOVA and binary logistic regression. *P <* 0.05 means significant difference.
