# Correction: Silence of cancer susceptibility candidate 9 inhibits gastric cancer and reverses chemoresistance

**DOI:** 10.18632/oncotarget.27523

**Published:** 2022-05-11

**Authors:** Chao Shang, Lin Sun, Jiale Zhang, Bochao Zhao, Xiuxiu Chen, Huimian Xu, Baojun Huang

**Affiliations:** ^1^Department of Neurobiology, College of Basic Medicine, China Medical University, Shenyang 110001, China; ^2^Department of Surgical Oncology, First Affiliated Hospital, China Medical University, Shenyang 110004, China; ^3^Department of Gastrointestinal Surgery, Dalian Municipal Central Hospital, Dalian 116033, China

**This article has been corrected:** Due to errors during image selection, incorrect images were chosen for the BCG823/BA - Control, BCG823/BA - pS-CASC9, and SGC7901/PA - Control representations in Figure 2E. The corrected Figure 2E, obtained using original data, is shown below. The authors declare that these corrections do not change the results or conclusions of this paper.

Original article: Oncotarget. 2017; 8:15393–15398. 15393-15398. https://doi.org/10.18632/oncotarget.14871

**Figure 2:**
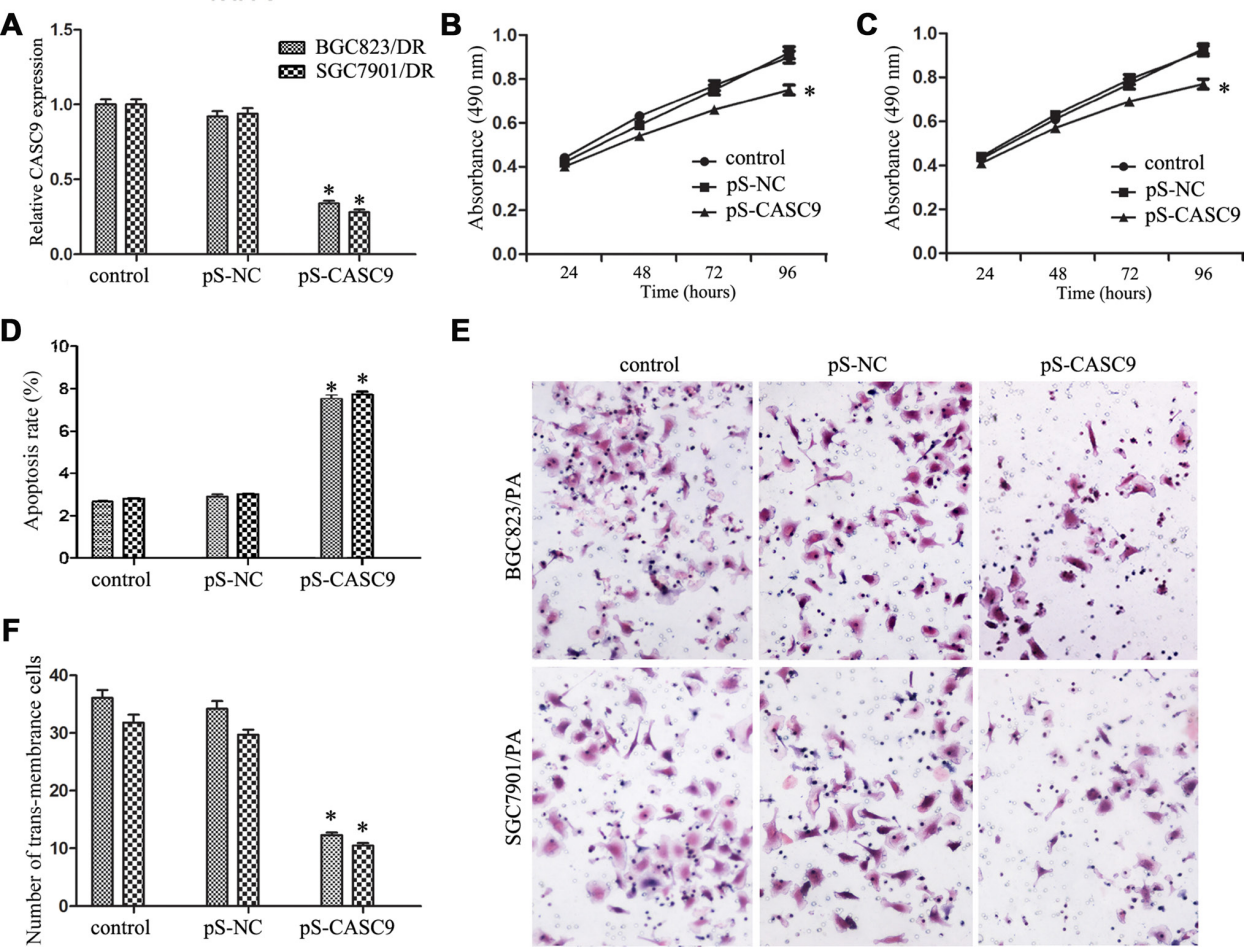
Knockdown of CASC9 inhibited proliferation and invasion.

(**A**) Expression level of CASC9 in BGC823/DR and SGC7901/DR cells after transfection of pS-CASC9. (**B**) Growth curve of BGC823/DR cells after CASC9 knockdown. (**C**) Growth curve of SGC7901/DR cells after CASC9 knockdown. (**D**) Apoptosis of BGC823/DR and SGC7901/DR cells after CASC9 knockdown. (**E**) Invasion assay by transwell analysis of BGC823/DR and SGC7901/DR cells after CASC9 knockdown. (**F**) CASC9 knockdown decreased the numbers of trans-membrance BGC823/DR and SGC7901/DR cells.

